# Fruit-Based Fermented Beverages: Contamination Sources and Emerging Technologies Applied to Assure Their Safety

**DOI:** 10.3390/foods12040838

**Published:** 2023-02-16

**Authors:** Alexandra Costina Avîrvarei, Liana Claudia Salanță, Carmen Rodica Pop, Elena Mudura, Antonella Pasqualone, Ofelia Anjos, Natalia Barboza, Jessie Usaga, Cosmin Pompei Dărab, Cristina Burja-Udrea, Haifeng Zhao, Anca Corina Fărcaș, Teodora Emilia Coldea

**Affiliations:** 1 Department of Food Engineering, Faculty of Food Science and Technology, University of Agricultural Sciences and Veterinary Medicine Cluj-Napoca, 400372 Cluj-Napoca, Romania; 2 Department of Food Science, University of Agricultural Sciences and Veterinary Medicine, 400372 Cluj-Napoca, Romania; 3 Centre for Technology Transfer-BioTech, 64 Calea Florești, 400509 Cluj-Napoca, Romania; 4 Department of Soil, Plant and Food Science (DISSPA), University of Bari Aldo Moro, I-70126 Bari, Italy; 5 Instituto Politécnico de Castelo Branco, 6001-909 Castelo Branco, Portugal; 6 Forest Research Centre, School of Agriculture, University of Lisbon, 1349-017 Lisbon, Portugal; 7 Spectroscopy and Chromatography Laboratory, CBP-BI-Centro de Biotecnologia de Plantas da Beira Interior, 6001-909 Castelo Branco, Portugal; 8 Food Technology Department, University of Costa Rica, Ciudad Universitaria Rodrigo Facio, San Jośe 11501-2060, Costa Rica; 9 National Center of Food Science and Technology (CITA), University of Costa Rica, Ciudad Universitaria Rodrigo Facio, San Jośe 11501-2060, Costa Rica; 10 Faculty of Electrical Engineering, Technical University of Cluj-Napoca, 400114 Cluj-Napoca, Romania; 11 Industrial Engineering and Management Department, Faculty of Engineering, Lucian Blaga University of Sibiu, 550024 Sibiu, Romania; 12 School of Food Science and Engineering, South China University of Technology, Guangzhou 510640, China; 13 Research Institute for Food Nutrition and Human Health, Guangzhou 510640, China

**Keywords:** fermentation, contaminants, mycotoxins, biogenic amines, pesticides, microplastics, heavy metals, decontamination

## Abstract

The food and beverage market has become broader due to globalization and consumer claims. Under the umbrella of consumer demands, legislation, nutritional status, and sustainability, the importance of food and beverage safety must be decisive. A significant sector of food production is related to ensuring fruit and vegetable conservation and utilization through fermentation. In this respect, in this review, we critically analyzed the scientific literature regarding the presence of chemical, microbiological and physical hazards in fruit-based fermented beverages. Furthermore, the potential formation of toxic compounds during processing is also discussed. In managing the risks, biological, physical, and chemical techniques can reduce or eliminate any contaminant from fruit-based fermented beverages. Some of these techniques belong to the technological flow of obtaining the beverages (i.e., mycotoxins bound by microorganisms used in fermentation) or are explicitly applied for a specific risk reduction (i.e., mycotoxin oxidation by ozone). Providing manufacturers with information on potential hazards that could jeopardize the safety of fermented fruit-based drinks and strategies to lower or eliminate these hazards is of paramount importance.

## 1. Introduction

With the expectation that there will be about 10 billion people on the planet by 2050, the pressure on the agrifood system to feed the world while staying within planetary boundaries has never been greater. Increased consumption of meat, fruits, and vegetables relative to cereals will be accelerated by wealth development in low- and middle-income nations, necessitating corresponding production changes and placing more strain on natural resources [1,2]. According to FAOSTAT, 2020 [3], global fruit production in 2018 was about 870 million metric tons (mt). The fruits with the most significant production are bananas (116 mt), watermelons (104 mt), apples (86 mt), and grapes (79 mt). Asia was the region with the largest global fruit production, with 490 mt. Latin America was the second largest region, with 133 mt, followed by Africa (109 mt) and Europe (76 mt), in global fruit production [3].

Fruit-based fermented beverages (FBFB) have been a pillar in human civilization for a very long time, thanks to their appealing sensory, dietary, and functional qualities. Fruits have a relatively high sugar content and include a significant number of bioactive substances, such as vitamins, antioxidants, and polyphenols, making them an ideal substrate for fermentation processes [4]. The most widely used FBFB around the world are wine and cider, but traditional beverages such as *hardaliye* [5], *tepache* [6] and *khadi* [7] are also gaining popularity.

The major problems in the beverage industry are chemical, microbiological, and physical hazards. Many pathogens occur across the food chain at various levels, because they are highly adaptive and can live, develop, and generate potentially hazardous substances in the microbiological environment [8]. Microbial populations are naturally present in raw fruits; therefore, the likelihood of exposure increases when raw materials are of poor quality [9]. Agricultural practices and climatic conditions are essential factors affecting the final product’s safety and quality. Climate change has significant implications for the chemical risks associated with FBFB consumption. Since the raw materials are susceptible to any minor change in climatic conditions, increased temperature results in an earlier harvest date and the appearance of non-specific contaminants for each region [10].

Food safety is of paramount importance in food processing. Hence, if good manufacturing practices are not properly followed, the processing of materials can represent a major source of contamination. Secondary sources of contamination in FBFB are presented after the packaging of the product and are related to the parameters of transportation and storage [11,12].

The contamination risk can be controlled through conventional methods, including chemical inhibitors, such as sulfur dioxide, which acts as a microbial inhibitor and antioxidant in FBFB. Many studies document intolerance or high sensitivity to sulfite additions, which can lead to a variety of negative side effects, including allergic reactions that increase the risk of asthmatic episodes, breathing difficulties, skin rashes, and stomach ache [13]. The use of high SO_2_ dosages must be avoided for both health and oenological reasons, since it might affect the finished product’s organoleptic properties, neutralize the aroma, and even result in recognizable olfactory flaws [14]. According to Tedesco et al. [15], a free sulfur dioxide dosage of 25–35 mg/L seemed to be efficient in eliminating viable *Brettanomyces bruxellensis* cells. Wells and Osborne [16] found a strong effect of bound SO_2_ on bacterial growth, which was bacteriostatic rather than bactericidal. The impact of bound SO_2_ on bacterial growth was always detected, and it was bacteriostatic rather than bactericidal. However, various yeast strains have developed tolerances to sulfite as a result of their genetic adaptation strategies [17,18]. Dimethyl dicarbonate (DMDC), a chemical preservative more effective against yeast than against bacteria or molds, prevents yeast growth in wines and disinfects musts by removing indigenous microbiota. In solution, DMDC may react with dimethyl carbonate, methyl ethyl carbonate, and methyl carbamate, resulting in compounds toxic to human health. Hence, the established maximum limit for DMDC in beverages is 250 mg/L [19].

Filtration is a physical process for removing microorganisms and is mainly used before bottling. Alcoholic fermented beverages are frequently filtered using filter aids, including diatomaceous earth, which poses a concern to public health, due to the presence of high quantities of heavy metals, such as arsenic, lead, and cadmium [20]. In terms of thermal processing methods, pasteurization and sterilization are most frequently applied. Their disadvantages include the loss of important bioactive compounds that affect appearance, taste, and nutritional value and lead to the deterioration of beverage quality [2,21].

Under emerging and constant environmental pressures, the food industries are obliged to endorse sustainable technologies that also meet efficiency and performance requirements. Artificial Intelligence (AI) and, in particular, machine learning (ML) can be used as tools for predicting and classifying features, automatically and without human intervention, leading to the detection and correction of abnormal actions or the improvement of environmental management. Blockchain technology has been shown to aid the food lifecycle, including safety, quality, and traceability [22]. Similar properties can be attributed to smart sensors that enable real-time monitoring and control of the production process. In addition, spectroscopy-based optical sensors have the ability to monitor not only the quality, but also the authenticity, of the food. The use of a cloud system is becoming more common, as it allows the processing of a massive collection of data in real-time at a low cost (e.g., reducing CO_2_ emissions in the beef supply chain). 3D printing has attracted a lot of attention in the last decade, due to advances in bakery, meat, and food packaging. Consumers’ personal preferences/needs can be used as requirements for developing customized food products, e.g., with specific nutritional value, texture, or absence of certain proteins (regarding people with allergies) [23].

Emerging technologies aim for new strategies to maintain or improve the quality and reproducibility of FBFB by controlling the microbial ecosystem. In order to control the fermentation stage, it is essential to choose the appropriate starter strain. Therefore, subsequent studies focus on the development of microbial starters [24], their improvement [25] and the efficient use of lactic acid bacteria (LAB) [26,27]. A wide range of yeast/bacterial strains are capable of inhibiting the action of biogenic amines [28], and multicopper oxidases have been identified in certain enzymes of *Lactobacillus plantarum* and *Pediococcus acidilactici* strains [29]. Pulsed electric field (PEF) is a non-thermal food preservation method that can inactivate enzymes and microorganisms within a short treatment period. Consequently, it can enhance the quality of beverages by conserving bioactive compounds and organoleptic properties [30]. The technology has been used for various matrices, such as pomegranate beverages [31], kombucha analogues [32], wine [33], and apple cider [34]. Ultrasonic treatment has been widely used in organic food processing to promote microbial proliferation to improve process performance, yield, and product quality. Ultrasound-assisted fermentation and extraction has been utilized in food processes in numerous studies regarding cider [35], a novel beverage from laver (*Porphyra dentata*) [36], wine [37], and beer [38]. High homogenization pressures (HPH) represent a physical process that allows the increase of a polydisperse liquid system’s particle size. Recently, HPH was applied in the wine industry, with promising results in minimizing the utilization of chemicals and maximizing the quality of wine production [39]. Carrot juice [40] and lupin beverage [41] had shown promising results regarding the shelf life and functional properties when HPH has been applied.

There are several tools and technologies available to reduce the risk of pathogens and contaminants and ensure the quality and safety of fruit-based beverages. The main approaches involve preventive measures in orchards, such as good agricultural practices (GAPs), good manufacturing practices (GMPs), and the implementation of additional steps for each process, such as chemical washing, high-pressure sprays, and pasteurization. Emerging technologies for the processing of fruits can comprise the use of pulsed electric fields (PEF), ultraviolet (UV), and high hydrostatic pressure (HHP) or ultrahigh pressure (UHP), as well as the adoption of HACCP systems to support these procedures [42]. Nevertheless, their application should prevent the loss of the peculiar aromatic notes and sensory profile, which could influence negative consumers’ perception [43].

**Figure 1:**
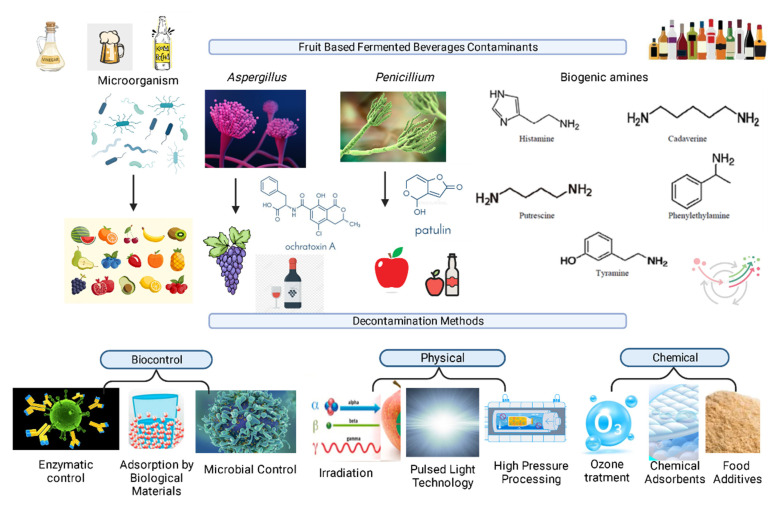
The main contaminants of fruit-based fermented beverages and the removal techniques.

Mycotoxin contamination in fruit fermented beverages is an ongoing concern for public health. Patulin (PAT), aflatoxins (AFs), and ochratoxin A (OTA) are the most prevalent mycotoxins that contaminate raw materials and derivates [44]. Mycotoxin exposure can be controlled in two ways: decontamination and preventive measures. Decontamination approaches used to completely deactivate or remove these high-toxicity mycotoxins include biological, physical, and chemical tools. Nevertheless, some techniques are less effective and occasionally prohibited because of concerns regarding safety, the potential loss of nutritional content of the treated products, and the expense of application [45]. Overall, it is important to consider the efficiency, processability, and cost of the decontamination methods, as well as the potential impact on the product. The principal contaminants of fruit fermented beverages and the removal techniques are shown in Figure 1.

This review provides an overview of the primary and secondary sources of contamination of FBFB, their associated chemical, physical, and microbiological hazards, and safety risks. Particular emphasis is placed on various emerging technologies and their limitations, with regard to the integration of these technologies into the food production sector and the supply chain to increase productivity and sustainability from a food quality and safety perspective.

## 2. Microbiological Risks Found in Fruit-Based Fermented Beverages

The development of microorganisms in alcoholic and non-alcoholic beverages depends on many important elements, including extrinsic and intrinsic factors. Intrinsic factors include the presence of antimicrobials, nutrient supplements, carbonation, and acidity. Nevertheless, the storage conditions, packaging, production method, cleanliness of the manufacturing facility, and raw ingredients will all affect the product’s microbiological quality [46].

Various microorganisms act as contaminants in beverages, but few can grow in an environment with low oxygen and acidity [46,47]. These are microorganisms typically associated with spoilage, a metabolic process that alters the sensory qualities of beverages to the point where they become unsuitable or unsatisfactory for consumption by humans [48], including a broad range of bacteria, molds, and yeast [49,50].

Traditional fermented foods and beverages do not often cause foodborne illnesses; however, this might be for a variety of reasons, including a low rate of persons seeking medical attention or flaws in foodborne disease surveillance systems [11]. However, bacteria can adapt to the acid and alcohol present in various fermented products, and most safety issues were involved by pathogenic bacteria, such as *Salmonella* spp. or *Escherichia coli* O157:H7 [11,46,51]. *E. coli* outbreaks were reported in cider and *Salmonella typhimurium* in orange juice [52].

Molds are naturally occurring contaminants of raw fruit and cause serious microbiological spoilage deterioration in fruit juices [53]. The mycelium or spores from the molds can infect the final product, byproduct, or raw materials. Molds that can harm fruit juice include species such as *Acremonium*, *Alternaria*, *Aspergillus*, *Aureobasidium*, *Botrytis*, *Byssochlamys*, *Cladosporium*, *Eupenicillium*, *Fonseceae*, *Fusarium*, *Geotrichum*, *Humicola*, *Monilia*, *Mucor*, *Neosartorya*, *Penicillium*, *Rhizopus*, and *Talaromyces* [11,46].

Yeasts are considered to be a common beverage contaminant. Owing to their exceptional ability to withstand carbonation levels above 3.0 vol. and acidic conditions, yeasts are recognized as an important class of organisms that spoil beverages. Their pH requirements range from 1.5 to 8.5, with 3.0 and 6.5 being optimal for growth [46]. One of the primary causes of contamination of non-alcoholic beverages is the ubiquity of the yeast population from raw materials. Variations in yeast density and diversity are caused by agricultural practices, varieties utilized, and the environment. Strains of *Cryptococcus*, *Rhodoturola* and *Sporobolomyces* are associated with aerial parts of plants and fruits, while *Candida*, *Debaryomyces* and *Pichia* are dominating the yeast community of fruit [54]. The spoilage of products appears when yeast cell counts reach 5 log CFU/g, and it becomes noticeable if it exceeds 7 log CFU/g [49,54]. Effects of spoilage include visible growth on the surface and fermentation in fruit juices, generated by yeasts like *Zygosaccharomyces balii* and *Pichia*. The yeasts *Dekkera/Brettanomyces* are a frequent cause of wine contamination, even in expensive wines. By producing 4-ethylphenol and 4-ethylguaiacol, *Dekkera* and *Brettanomyces bruxellensis* yeasts give wine an unsavory off-flavor that is commonly referred to as “Brett character” and defined as “leather”, “horse sweat”, “stable”, and “smoke” [55]. The haze, production of organic acid, and odd flavors in wine are generated by *Brettanomyces anomalus* [49].

The most widely identified concern regarding strong and weakly fermenting species is spoilage accompanied by excess gas (carbon dioxide), in species such as *Z. bailii*, *S. cerevisiae* (var. *diastaticus* is a feared contaminant for bottled lager beer [56]), *D. bruxellensis*, *Saccharomycodes ludwigii* or *C. parapsilosis* and *Candida pseudointermedia*, respectively [49]. *Zygosaccharomyces* genus is considered the most frequent spoilage yeast and is responsible for considerable economic loss in the beverage industry [54].

## 3. Chemical Risks Found in Fruit-Based Fermented Beverages

### 3.1. Mycotoxins

Mycotoxins are secondary metabolites of filamentous fungi, such as Aspergillus, Fusarium, Penicillium and Alternaria, and occur ubiquitously in the food chain [57]. The prevalence of these compounds in products such as fruits, cereals, vegetables, beverages, and other agricultural products emerged as a serious issue for human health, due to their ability to produce severe toxic effects (carcinogenic, genotoxic, teratogenic, nephro- and hepatotoxic) [58].

About 25% of agricultural communities worldwide are contaminated with mycotoxins derived from saprophytic or endophytic fungi [59]. At the time of writing, more than 100 fungi have been reported to produce several hundred fungal metabolites with toxigenic potential. Mycotoxins frequently found in food and feed include AFs, OTA, deoxynivalenol, zearalenone, fumonisins, and patulin, which can be produced by various fungal species [60].

Mold growth and mycotoxin formation is an accumulative process and can emerge at several points throughout the food chain, which may begin in the field and intensify during subsequent phases, such as harvest, drying, and storage. The main factors affecting mycotoxin formation are temperature, water activity (aw), relative humidity (RH), pH, fungal strain, and substrate [61].

Humidity and temperature are closely related and have a critical impact on mold and mycotoxin production. The optimal temperature for the production of mycotoxins by fungi is between 20–30 °C. In regions with tropical and subtropical climates, aflatoxins B1, B2, M1, M2, G1 and G2 are more common, while, in temperate climates, fusariotoxins, e.g., trichothecenes, are most abundant [62].

Medina et al. [63] studied the link between temperature and water activity (a_w)_ of Fusarium verticillioides and the mycotoxins fumonisin (FB1 and FB2). The study found the optimal temperature range for the growth of F. verticillioides strains was 20–25 °C and a_w_ of 0.995. However, for FB1 and FB2 production, the optimal conditions for a_w_ were 0.98–0.995 and 20 °C or 20–30 °C for temperature, respectively. Consequently, the environmental factors required for mycotoxin production differ from the growth factors. Another study [64] reported that AF contamination was more pronounced at 90% RH (3.9 μg/kg–11,179.7 μg/kg) than at 60% RH (0.3 μg/kg–2.4 μg/kg). However, moisture content was the only factor that did not create a discernible impact on AF content. As for temperature, AF occurred at 20 °C and 30 °C, with the indication that contamination was higher at 30 °C.

Microorganisms have the ability to adapt to changes in the environment, and pH affects the development of fungi and their mycotoxins. Molds may secrete acids or alkalis (butyrate, oxalate, malate, citrate, gluconate, and succinate) to enhance their virulence by locally reducing the pH of their host [65]. For example, Sclerotinia sclerotiorum and Botrytis sp. produce gluconic acid, oxalic acid, or citric acid, the role of which is still controversial and likely involves suppression of plant defenses, triggering programmed cell death and deregulation of guard cell function [66]. The expression of the biosynthetic genes can also be impacted by pH. The optimal range for Alternaria mycotoxin production is 4.0–4.5, while pH above 5.5 inhibits formation [67].

Mycelial growth and mycotoxin generation are affected by osmotic pressure in the substrate. The optimum temperature and osmotic pressure for *Fusarium proliferatum* growth were found to be 28 °C and −51.02 bar, respectively. Growth is limited by low or high osmotic pressure ranges [68]. To sustain development and provide energy, filamentous fungi have the innate capacity to hydrolyze a variety of carbon sources. Aspergillus niger can utilize sugars as the sole source of carbon and energy for cell development and metabolism. It is widely known that A. niger grows and distributes itself in response to saccharides, generally including whole colony proliferation, biomass increase, and reduction of the carbohydrate content in the environment [69]. Consequently, the presence of fungi does not inevitably indicate eventual mycotoxin contamination, as the parameters required for their formation are distinct from those that encourage fungal development. Due to their chemical stability and thermostability during food processing, the elimination of fungus from food does not ensure the absence of mycotoxins [70].

Environmental stresses, such as insect infestation, drought, mechanical damage, nutrient deficiencies, erratic temperatures, precipitation, or humidity, can facilitate mycotoxin formation in growing crops. Good agricultural practices reduce plant stress, thereby reducing fungal invasion and mycotoxin contamination [71].

Later studies reported the presence of mycotoxins in beverages, especially fruit- and vegetable-based beverages (e.g., wine and fruit juice), beer as a cereal-based beverage, and milk as an animal-derived product. In FBFB, the cause of contamination is the poor quality of the raw material and its production. PAT, OTA, and Alternaria toxins are the most relevant and frequent mycotoxins in fruits and their processed products [72].

FBFB, such as cider and wine, are consumed in large quantities worldwide; hence, their contamination represents an important food safety issue [73].

OTA is reported in wine primarily due to contamination of grapes by *A. carbonarius* and *A. niger* while still in the plantation or in the phases before vinification. In dietary exposure assessment, the European Commission informed that wine ranks as the second most important food, with the highest contribution to the daily intake of OTA by the EU population, resulting in a maximum allowable level in wine of 2 µg/kg [74]. Although OTA is reduced up to 80% throughout the winemaking process, modified mycotoxins may be formed [75]. In fact, modified mycotoxins usually remain undetectable in conventional analyses. Studies have shown that the total content of mycotoxins in wine is usually underestimated, due to the formation of OTA derivatives [76,77].

The primary producer of PAT is the fungus *Penicillium expansum*, which causes blue mold disease in apples. Despite regulations, studies report high levels of PAT in commercial beverages, sometimes exceeding the limits. Good agricultural practice (GAP) and good manufacturing practice (GMP) are the most effective means of limiting fungal growth and metabolite production. In apple cider, PAT is degraded by yeast action during fermentation. Al Riachy et al. reported that, after 2 days of fermentation, the content of PAT in contaminated must decreases sixfold [78]. Thus, the presence of patulin in cider is primarily due to the addition of apple juice in specific industries, such as sweet or low-fermented cider. Nevertheless, there are still cases where the level of PAT in commercial ciders exceeded the limit of 50 g/L [79].

*Alternaria* species produce the dibenzo-pyrone mycotoxin referred to as alternariol. Chronic exposure may have mutagenic, carcinogenic, xenoestrogenic, and immunomodulatory effects. Alternariol (AOH) contamination has been reported in several products, such as cereals [80], chestnuts [81], oilseeds [82], and fruits [83]. Soft-skinned fruits, such as grapes and tomatoes, and their products are often susceptible to *Alternaria* infection and are, therefore, frequently contaminated with AOH. Carballo et al. [84] reported that AOH was the most prevalent mycotoxin in 90% of the beer samples, with an average value of 19.39 µg/L. In addition, 10% of the cider samples reached 21.56 µg/L and 93% of samples in red wine had a level of 7.7 µg/L.

### 3.2. Biogenic Amines

Biogenic amines (BA) are nitrogen-containing compounds with a low molecular weight, formed by enzymatic reactions, such as decarboxylation, transamination, reductive amination, and degradation of the corresponding precursor amino acids [85]. The chemical structure can be classified as aliphatic (cadaverine, putrescine, agmatine, ornithine, spermidine, and spermine), aromatic (β-phenylethylamine and tyramine), or heterocyclic (tryptamine and histamine) [86].

The BA emergence in foods and beverages is attributed to the availability of proteins and/or free amino acids, which are the substrate for natural enzymes of the raw materials or are produced by microbial decarboxylation or amination activity [87]. Microorganisms such as *Escherichia*, *Enterobacter*, *Pseudomonads*, *Salmonella*, *Shigella*, *Clostridium perfringens*, *Streptococcus*, *Lactobacillus* and *Leuconostoc* are capable of producing specific microbial metabolites (e.g., histamine, tyramine, putrescine, cadaverine and β-phenylethylamine) commonly related to food hygiene and technology, while, in the case of LAB, the presence of BA indicates a defense mechanism during fermentation [88].

Several characteristics play a role in their development, which can be classified according to the raw materials (e.g., presence of NaCl, pH, redox potential, or water activity), microorganisms (decarboxylase activity is primarily linked to *Escherichia*, lactic acid bacteria (LAB) *Streptococcus*), and processing and storage conditions (e.g., fresh, cured, fermented, modified atmosphere) [89].

A high percentage of BA in the final product is often associated not only with a high number of decarboxylating cells. The optimal temperature promotes cell metabolism and proliferation of BA. According to the European Food Safety Association (2011), BA is produced by mesophilic bacteria in significant amounts between 20 and 37 °C, while the production of BA declines below 5 °C or above 40 °C [90]. A study conducted by Jirath et al. [91] showed that *Klebsiella pneumoniae* in meat is a large producer of cadaverine at temperatures above 20 °C. Psychrotolerant bacteria have a high contribution toward the amine accumulation at temperatures below 5 °C. For example, *Photobacterium psychrotolerans* and *Photobacterium phosphoreum* produce histamine in seafood at storage temperatures of 0–5 °C [92].

The pH is correlated with two mechanisms that act simultaneously and whose result depends on their balance. The first is related to acidity in food, which acts as a barrier to the growth of microorganisms [93]. Bacteria exhibit decarboxylase activity as part of their defense mechanism against lower pH [94,95].

The final concentration of BA in foods and beverages is not characterized by the isolated effect of a single factor, but by several combined effects. As a result, these metabolites are extremely difficult to destroy by further processing steps (e.g., pasteurization, cooking, etc.) [96]. Due to their toxicity, BAs can be considered a quality marker, as it is indicative of product freshness and food safety. More specifically, elevated levels of certain amines in foods can be attributed to the use of raw materials, with poor quality, contamination, or improper conditions during storage or processing [97].

Consumption of large amounts of BA has been implicated in several instances of food poisoning and is correlated with a wide range of toxicological and health risks that include psychoactive [98], vasoactive, and hypertensive effects [99].

In fruit-based fermented beverages (FBFB), the formation of BAs is related to the amino acid content, variety and ripeness of the fruits, as well as the techniques used. Moreover, it could occur naturally in the raw material or as a result of the various stages of production and storage. However, several stress factors, such as intensive nitrogen fertilization, pest infestation, mold infestation, and other parameters affected by climate or soil type and composition, have a large influence on the BA content [100].

Studies have shown that wines derived from vines treated with nitrogen fertilizer may have toxicity problems due to the content of BA [101]. For example, Ancín-Azpilicueta et al. [102] demonstrated that foliar urea significantly increases the concentrations of histamine and spermine (in the range of 8–20 mg/L) in wine, which may have toxic effects for consumers.

During the winemaking process, the main processes that favor the formation of BA are primary fermentation by *Saccharomyces cerevisiae* yeast and the malolactic fermentation of LAB. Nowadays, the most representative BA found in wine are histamine, tyramine, putrescine, cadaverine, and phenylethylamine. For example, putrescine has been accumulated in grapes as a response to a potassium deficiency in soil [103]. Moreover, red wine reported higher concentrations of BA than white wine, attributed to the prevalence of malolactic fermentation and the extended maceration with grape skin, which produces large amounts of polyphenols and free amino acids [104].

Ouyang et al. [105] analyzed a range of fermented beverages made from various fruits, such as raspberry, strawberry, blackcurrants, plums, goji berry and grapes. Red grape wines had the highest total biogenic amines content (28.11–67.48 mg/L), followed by strawberry wine (14.60 mg/L) and raspberry wine (8.75 mg/L). The total content in white grape wines ranged from 5.42 to 7.21 mg/L, while the other fruit wines showed a level below 2.5 mg/L.

So far, the regulations in force in the EU do not concern beverages, but only histamine in fishery products [106]. According to EFSA, only a few European countries have arbitrary established legal limits for histamine in beverages (wine and beer): 2 mg/L in Germany, 6 mg/L in Belgium; 8 mg/L in France; 4 mg/L in the Netherlands; and 10 mg/L for Switzerland and Austria [90].

### 3.3. Pesticides

The use of pesticides on agricultural land is related to the increasing demand for food. To increase crop productivity, pesticides are widely used to control pests in fruits and vegetables [107]. A pesticide is a natural or synthetic substance, or a mixture of substances, used to prevent, destroy, or spread disease. Due to their different chemical and physical properties, they are classified according to their main use (such as insecticides, herbicides, and fungicides [108].

Pesticide application on agricultural land is related to the increasing demand for food. To increase crop productivity, pesticides are commonly used to control pests in fruits and vegetables [107].

Pesticides can also be divided into organic pesticides and inorganic pesticides. Organic pesticides are further divided into two groups: natural organic (natural sources such as plants: rotenone, pyrethrum) and synthetic organic or modern (artificially produced by chemical synthesis: dichlorodiphenyltrichloroethane, permethrin, malathion, lindane, etc.) [109]. Among the different groups, organophosphates and carbamates are the most effective and commonly used pesticides. They are known to act on the neurological system of pests, causing paralysis and eventual death of the organism. In addition to their ability to kill insects, they have also been reported to inactivate acetylcholinesterase in humans, causing acetylcholine to accumulate, leading to convulsions, seizures and even death [110,111].

The behavior of pesticides is determined by various factors, such as temperature, light, humidity, bacteria, and pH, causing them to break down at different rates [112]. Pesticide resistance and climate change are two of the greatest problems facing today’s society. The volatilization of pesticides is an undesirable process, not only from an economic point of view (the effect is not achieved, hence more pesticides have to be applied) but also due to the pollution of the atmosphere and the exposure of humans and other plants to the effect [113,114].

Temperature affects the evaporation of pesticides by changing the vapor pressure and volatility. The effectiveness of azoxystrobin varies spatially and is highly influenced by air temperature [115]. Organophosphates, carbamates, synthetic pyrethroids, and sulfonylureas are hydrolyzed more rapidly when exposed to higher temperatures [116]. For example, chlorpyrifos was degraded faster at 24 °C than at 20 °C, resulting in decreased mortality and oxidative damage to insect pests [117].

The humidity effect on pesticide volatilization can be defined through the mechanism of sorption on mineral surfaces under dry conditions. The sorption process correlates strongly with water activity, expressed as equilibrium-relative humidity in the pore space of the soil, and with the available surface area of hydrated minerals [118]. Therefore, humidity may increase degradation through hydrolysis for susceptible pesticides. Schneider et al. (2013) [119] demonstrated that triallate and trifluralin had considerably increased volatilization at 60% RH (7% for trifluralin and 6% for triallate) than at 90% (47% and 32%, respectively).

The ionic character of some pesticides, the magnitude and nature of the charge of the soil medium and/or organic matter, and the route that plant roots take to absorb the substance are all affected by soil pH, which also changes the harmful effects of some pesticides on plants [112]. The relationship between pH and degradation rates depends on the predominant degradation method of each pesticide. Experimental studies have shown that imidacloprid and fipronil from paddy water increases the process of degradation at pH 10 after 44.7 days and 13.2 days, respectively [120].

The most common pesticides found in wine included azoxystrobin, boscalid, cyprodinil, dimethomorph, fenhexamid, fludioxonil, and metalaxyl. During winemaking, pesticide residues are transferred from the grape skin to the must and further into the wine, which may pose a toxicological risk to the consumer. The residues can be absorbed by the solids produced during fermentation or lost during refining. In addition, red wine may have elevated levels of pesticides, due to the long maceration (which involves the grape skin) [121]. Čuš et al. (2022) detected several pesticide residues (dimethomorph, iprodione and pyrimethanil) in organic wines, which are prohibited for this particular type of production [122]. Unquantifiable residues of parent pesticides (below the limit of quantification of 0.01 mg/kg) are most frequently found in wine. However, the presence of these metabolites represents a concern for food safety and quality [123].

In addition to their harmful effects on human health, pesticides also have a major impact on wine flavor. Song et al. (2022) and Russo et al. (2019) reported that *S. cerevisiae* growth was significantly inhibited by these five pesticides (hexaconazole, difenoconazole, flutriafol, tebuconazole, and propiconazole) at the maximum residue level [124,125]. During winemaking, 86 altered metabolites were detected, resulting in a significant change in the fermentation profile of the yeast.

Fungicides are a major problem in apple production. Studies have indicated that fenbuconazole residues, even at low concentrations (0.2 mg/L), are of concern in cider production, due to their effects on the fermentation rates [126]. However, other insecticides, such as pyridaben, have been shown to accumulate primarily on apple peels, making peeling, coring, and juicing an effective solution that can reduce residues by up to 90% [127].

Regardless of their advantages (e.g., easy availability and low cost), they have hazardous effects, ranging from short-term (e.g., skin and eye irritation, headaches, dizziness, and nausea) to chronic (e.g., cancer, asthma, leukemia, and diabetes) conditions [128]. There is also evidence of the adverse effects of pesticide exposure leading to congenital disabilities, lower birth weight, fetal death, etc. [129,130,131]. The risk depends on several factors, such as the period and level of exposure, the type of pesticide, and the environmental characteristics of the affected areas. However, there is no population group that is not exposed to pesticides [132].

The World Health Organization (WHO) estimates that, each year, between 0.4 and 1.9% of persons die as a result of acute poisoning from pesticide contact, which affects approximately 1,000,000 [133].

A joint committee between the Food and Agriculture Organization (FAO) and World Health Organization (WHO) was established to coordinate beverage standards as well as food standards, and it has established an international code of conduct for pesticide management. The code provides the best practices for managing pesticides throughout their life cycle, from manufacturing to disposal, for government regulators, the commercial sector, civil society, and other stakeholders. They advocate good agricultural practices for the responsible use of pesticides [109].

### 3.4. Heavy Metals

Recently significant interest has been focused on the investigation of the metal content of foods and beverages [134]. Heavy metals are naturally occurring elements with high atomic numbers; most heavy metals occur in the Earth’s crust, where they are entangled with various natural and anthropogenic activities. Heavy metals cannot be easily degraded [135]. Heavy metals are classified as essential and non-essential [135]. Certain metals, such as Cu, Fe, and Mn, are essential for human life, and they are essential natural substances for development and respiration [136]. However, when the concentration surpasses the tolerated limit for organisms, essential heavy metals can be hazardous to living beings [135].

Heavy metal contamination of food and beverages is frequently the result of environmental [135] and industrial contamination, such as equipment used for fermentation, conditioning, filtration, carbonation, and packing. Additionally, water, raw materials used, storage or ageing, and equipment/utensils used are important sources of metals that find their infiltration into food and beverages [137]. The sources of heavy metals contamination in beverages are classified as exogenous sources (heavy metals that come from chemicals added during production and contamination from industrial equipment used for fermentation, conditioning, filtration, carbonation, and packaging) and endogenous sources (natural components like water, yeast, and raw materials, such as cereals, used in the manufacturing process) [138].

Beverages serve a crucial role in sustaining life and, when contaminated, have the power to spread many diseases. Drinking both alcoholic and non-alcoholic beverages is a significant way that heavy metals can enter the body [135]. The report by Abdel-Rahman et al. (2019), where heavy metals were determined in Egyptian non-alcoholic beverages, revealed that the Pb, Cd, and Cr were non-detectable, while Cu content varied between 0.17 and 0.56 mg/kg; Fe content was 43.88 mg/kg; Ni was 0.53 mg/kg and Mn was 1.24 mg/kg. Still, the results indicate that the average concentrations of Cu, Cr, Pb, Mn, and Cd in all non-alcoholic beverage samples were within the maximum permissible limits of metals in beverages, stated by the WHO and the Egyptian Ministry of Health (EMH) reports for drinking water [136]. The source of the raw materials, the water used to dilute the juices, the techniques employed for fruit cultivation, and contamination of the processing equipment when materials other than stainless steel are used are all factors that might cause FBFB heavy metals contamination.

A survey of metal profiles in some traditional alcoholic beverages (oil palm wine, *raphia* palm wine, *burukutu*, *pito*, *ogogoro*) in Nigeria was determined by Iwegbue et al. (2014) [139]. The results indicate that the mean concentrations of the metals varied significantly, depending on the analyzed metal, and were below statutory limits for the metals in alcoholic beverages: Cd (0.02–0.05 mg/L); Pb (0.01–0.19 mg/L); Ni (nd−0.11 mg/L); Cr (nd−0.15 mg/L); Cu (0.09–0.60 mg/L); Co (0.01–0.08 mg/L); Fe (0.30–10.3 mg/L); Mn (0.02–3.97 mg/L); and Zn (0.12–3.84 mg/L). Osei et al. [140] examined the presence of heavy and trace metals in two non-alcoholic drinks (*asaana* and *nmedaa*) and two alcoholic drinks (*burukutu* and *pito*) made in Ghana. All four drinks contained concerning levels of Ni, Zn, and Pb; pans and utensils, as well as the water supply used to make these drinks, were identified as the most likely sources of these metals.

Cu and Zn were shown to be the most widespread pollutants via process water, manufacturing equipment, and packing devices, according to heavy metal analyses [141].

The daily dietary intakes of heavy metals are determined by the legal authorities, who also regularly update them. For example, cadmium (0.36 µg/kg body weight) [142], is a very present heavy metal contaminant in fruits and vegetables, due to its easy soil-to-plant transfer [143], and it is also present in the packaging materials and ceramic food contact articles [144]. Despite the fact that each food or beverage consumed separately is not concerning, high volumes of contaminated drinks and prolonged exposure to heavy metal consumption may create physical, muscular, and neurologically degenerative sickness issues [135]. Therefore, it is very essential to follow a food safety system during the manufacturing of non-alcoholic and alcoholic beverages [136], in order to prevent elevated levels of toxic metals by regularly checking the water and raw materials, as well as the processing procedures [135].

## 4. Microplastics Detected in Fruit-Based Fermented Beverages

Microplastics have become an actual environmental pollution problem with many implications for animal and human health [145]. Microplastics (MPs) are typically defined as particles of sizes between 1 μm and 5 mm that include a variety of chemical components originating from multiple sources.

Due to their strong hydrophobicity, particle size, high specific surface area, stable chemical properties, and capacity to transport additional environmental pollutants (such heavy metals and antibiotics), microplastics possess the capacity to accumulate, migrate, and spread in the environment [146].

Recent studies have detected microplastic fibers and particles in some food groups [147], including honey, soft drinks, cold tea, energy drinks, beer [148,149], and seafood [150].

Three major pathways make food products susceptible to microplastic contamination. Due to its small size, MPs are easily swallowed by marine and terrestrial creatures (such as fish, mussels, crabs, and poultry) and absorbed by plants, eventually entering human bodies through the food chain. In addition, plastic is a material that is frequently used to package foods [145].

Food and beverages are the main potential sources of MP exposure to dietary contamination. The microplastic contamination in drinking water leads to the contamination of beverages [145].

MPs (fibers 2–79 MPs/L) were discovered in each of the 24 beer brands analyzed by Liebezeit et al. [149]. Additionally, MPs were found in all the blank (double-distilled water) samples [149]. Shruti et al. [148] investigated the microplastic abundance in 13 Mexican beers and 19 samples of soft drinks. MPs were found in all the samples; for beer samples, the values were 0–28 MPs/L, and for soft drinks, values were 0–7 MPs/L. Compared to Mexican beers, German beers had the highest number of contaminated samples. However, the number of MPs discovered may vary as a result of the various detection techniques applied [148].

In their research, Cox et al. [151] proved that male children and adults consumed an approximate daily MPs intake of 113 particles, 142 particles, 106 particles, and 126 particles, respectively, from food and beverages.

Assessing the microplastic contamination and their distribution in food and drinks is useful for determining how microplastics are affecting various food products, what plastic materials impact contamination, and how to implement elimination actions [148].

## 5. Techniques Applied for Limiting Formation and Contaminants Removal

### 5.1. Enzymatic Procedures

The biological methods of decontamination using microorganism control (bacteria and fungi) or their enzymes evolved after the 1960s. In the last decade, studies have focused on the use of bacteria for mycotoxin decontamination and the proposed decontamination mechanisms. In total, 33 species have been investigated, from *Alcaligenes*, *Bacillus*, *Brevibacterium*, *Cupriavidus*, *Devosia*, *Escherichia*, *Enterobacter*, *Lysinibacter*, *Lysinibacillus*, *Pediococcus*, *Pseudomonas*, *Rhodococcus*, and *Streptomyces*, as well as lactic acid bacteria. Over the years, the literature reported various microbial enzymes from yeast, bacteria, and fungi capable of mycotoxin detoxification and transforming AFs from wine and grape juice into less or nontoxic metabolites [152], such as *Oenococcus oeni* [153], *S. cerevisiae* [154], *Candida famata* [155], and *A. niger* [156].

The literature identifies two primary methods of microbial decontamination: adsorption to the chemicals found in cell walls (peptidoglycan, glucomannan, and -D-glucan) and biotransformation to less toxic or non-toxic compounds through the production of appropriate enzymes [45]. Enzyme applications is one of the greatest impacts of biotechnology on the food segment and represent a successful tool in solving processing issues in order to improve the quality and safety of fruit-fermented beverages using a cost-effective method [157].

Generally, enzymatic processing techniques are used to improve limpidity and shelf stability with lower viscosity and turbidity of the products [158]. The quality of grape wine and other fruit fermented beverages is strongly linked to the application of enzymes in the technological process. The effects of porcine pancreatic lipase (PPL) immobilized in calcium carbonate on the degradation of PAT in apple juice was investigated by Tang et al. [159]. The PAT degradation rate was over 70%, and the optimum degradation conditions of PAT were 0.03 g/mL immobilized PPL for 1 mg/L patulin, 40 °C and 18 h [159].

By adsorbing into their cell walls, certain microorganisms have the capacity to extract mycotoxins from the food matrix. About 20–90% of mycotoxins could be adsorbed by gram-positive bacteria and yeasts in the different liquid food systems. Due to their beneficial effects on human health and the environment, biological materials, such as microbial cell walls, peptidoglycans, chitosan, chitin, and enzymes, are favored for use in the food sector. However, their drawbacks include a lower degree of effectiveness and higher costs compared to chemical and physical methods [44].

In the study conducted by Castro et al. [160], *Lactobacillus plantarum* was encapsulated in a polymeric matrix composed of polyvinyl alcohol and alginate. The results revealed that a concentration of 0.5 g/mL of the complexes formed removed over 50% of the OTA from contaminated red wines [160]. Farbo et al. (2016) indicated that yeast cells have a strong potential for usage as a reliable and secure technique to extract OTA from liquid matricest [161]. Zhu et al. [162] observed that, after 2 days of *Rhodosporidium paludigenum* incubation at 28 °C, the PAT level in nutritional yeast dextrose broth with 10 mg/L had been reduced by 100%. These results reveal a way to use with *Rhodosporidium paludigenum* and its purified enzyme for the detoxification of patulin in fruit-derived products [163]. When autoclaved *Candida intermedia* cells were added to the grape juices, either free-floating or bound in magnetic alginate beads, the concentration of OTA was significantly reduced by almost 80% from its initial level [161].

### 5.2. Chemical Methods

Chemical practices comprise methods such as hypochlorite treatment, ammoniation, ozone treatment, alkaline hydrogen peroxide treatment, chemical adsorbents, and food additives to detoxify mycotoxins in alcoholic beverages [44,164]. Treatment with chemicals can efficiently destroy the structure of the mycotoxins by using strong oxidants, acid, base and other chemical substances. Among them, ozone has the ability to oxidize the double bond of mycotoxin structure and forms products with less toxicity and, thus, is applied to degrade PAT, OTA and trichothecene to improve the safety of beverages. Ozone is preferable to other chemical oxidizing agents, due to the fact that is available in both gaseous and aqueous form for application; numerous ozone precursors exist; there are no traces (residue); there is no related hazardous disposal; and onsite ozone production is possible [165]. Diao et al. [166], reported that 10 min of exposure to gases containing 7 and 12 mg/L of oxygen decreased the PAT concentration in apple juice by 64.77 and 81.66%, respectively [166]. Additionally, ascorbic acid (vitamin C), sulfur dioxide, thiamine (vitamin B1), vitamin B6 and calcium pantothenate were successfully used for degrading mycotoxins. However, these substances are restricted by juice manufacturers, owing to the influence on the nutritious components of the juice [167]. Mycotoxins are absorbed via the use of chemical adsorbents, such as sulfhydryl-terminated magnetic beads, magnetic carbon nanotubes (Fe_3_O_4_-MWCNTs), and propylthiol-functionalized SBA-15 silica [44,168]. Clay, cholestyramine, esterified glucomannan, activated charcoal, and other modified polymers are utilized as adsorbents, and they can absorb mycotoxin in liquid environments from 17% and 100%. There is no current chemical technique for OTA degradation in wines [169]. A recent study presented the effectiveness of already documented wine-refining substances, including bentonite, chitosan, potassium caseinate, activated carbon, and the elimination of aflatoxins B1 and B2 from both white and red wines. At a level of 120 g/hL, bentonite was the most effective fining agent, eliminating nearly all aflatoxins from both white and red wines [170]. The presence of biogenic amines (BA), like histamine, putrescine and cadaverine, is very frequent in fermented products due to microorganism metabolism. Wine biogenic amine removal from wines using various functionalized silica materials (cation-exchange materials) was assessed [171]. That mesoporous silica material bifunctionalized with phosphonic and sulfonic acids enabled the removal of histamine, putrescine, cadaverine, spermine, and spermidine from wines; however, the dose adjustment was necessary for accordance with the removal requirements and the initial levels in the wines. Bettini et al. [172] have studied paramagnetic iron oxide nanoparticles that have been synthesized and covered by silica shells, manufactured for two purposes: SiO_2_ capping enhances the nanoparticles’ stability while also boosting the interaction between biogenic amines and paramagnetic nanoparticles @SiO_2_ (MNPs@SiO_2_). The biogenic amines were removed efficiently from wine with the aid of weak magnetic fields. One of the most affordable detoxification techniques is the use of chemical adsorbents. Nevertheless, there are many safety concerns about their use regarding adverse health effects, due to the possibility of release of hazardous substances from chemical adsorbents to beverages. Additionally, the sensorial profile and quality parameters can be adversely affected by chemical adsorbents [44]. In the study by Kim et al. (2018), PAT level was measured in apple juice treated with citric acid, sodium bicarbonate, vinegar, a mixture of sodium bicarbonate and vinegar, baking powder, and ultraviolet (UV) irradiation. Among these food-grade additives, sodium bicarbonate (from 94.11 to 7.55 µg/L) and UV (30 min) irradiation had the greatest impact in lowering PAT [173]. Mohammadi and Ziarati [174] studied the citric acid’s chelating and pH-adjusting abilities to allow for the most effective removal of heavy metals, including cadmium, nickel, and lead from fruit juice products. Another benefit of the suggested procedure is that the complex created by citrate has a clear crystalline structure and is simple to remove using a centrifuge or filter [174].

Chemical methods are simple to use and relatively affordable, but their primary drawback is the toxicity of their residues and byproducts.

### 5.3. Physical Procedures

Physical detoxification methods suggest the use of physical adsorption, filtration through micropore membranes, microwave, UV, gamma irradiation, thermal treatment, pulsed light technology (PL), and high-pressure processing (HPP) for reducing the mycotoxin concentration in beverages [44,167,175]. Mycotoxins present a high resilience to degradation by thermal treatments, such as pasteurization and distillation. Wine filtering using a 0.45 mm membrane decreased up to 80% of OTA [169]. Sun et al. [176] reported the ability of proteins to remove OTA at a proportion of 80–94% from Chinese red wine after treatment with 0.20 mg/mL egg and centrifugation for 48 h [176]. For the removal of mycotoxins from fruit beverages, the most common adsorbent materials capable of effectively encasing and immobilizing the mycotoxin are activated carbon, microporous resin, and diatomite [177,178]. In the study conducted by Pramanik et al. [179], microwave-treated PAT in apricot juice for 15 min results in a 95% decrease, with no significant changes in sensorial profile [179]. Currently, due to consumer concerns, irradiation technology is not frequently used in the food sector. However, UV irradiation demonstrated good efficiency in the degradation of PAT in apple juice and cider [162,180] (Table 1). In order to minimally alter the quality of fruit juices, Kalagatur et al. [181] conclude that the degradation of mycotoxins in fruit juices might be facilitated by irradiation at doses up to 10 kGy [181]. Parameters such as temperature/time influence the degradation of mycotoxins. Thermal treatment can be combined with HPP to accelerate the degradation of mycotoxins in products. HPP is an emerging non-thermal food-processing technology, using 100 MPa to >1000 MPa for short periods, and has several benefits over traditional thermal processing techniques, including retaining the freshness, flavor, texture, appearance, and color and reducing the loss of the nutritional value of the products. Currently, HPP is commonly utilized as a non-thermal food technique to pasteurize fruit juices and beverages [182]. Hao et al. [183] demonstrated the reduction of PAT by HPP treatments of 600 MPa up to 31% in fruit juice blends. Additionally, the treatment of apple juice with HPP has been reported to lower the PAT concentration by up to 51% [184]. An emerging technique for non-thermal food processing is pulsed light (PL) which uses short, high-intense light pulses to remove mycotoxins from the product [44]. Compared with conventional tools, PL minimizes the deleterious effects and preserves nutritional and sensorial properties [185]. In the study by Funes et al. [186], apple juice and apple purée treated with PL doses of 2.4 and 35.8 J/cm^2^ ensure an increase in PAT of 22% and 51%, respectively. Physical methods can be applied in the beverages industry, but with some limitations regarding irradiation, which has a negative impact on a product’s nutritional value, antioxidant, and sensorial attributes. HHP and LP can be considered risk management tools to increase levels of mycotoxins in beverages.

### 5.4. Biological Decontamination Procedures

Decontamination using microorganisms has many advantages, such as effectiveness against different mycotoxins, being environmentally friendly, free of chemicals, and adsorption by dead biomass, and the possibility to be used during the fermentation stage, but there are still some limitations. These include limited implementation and low potential as a method of detoxification when applied to foods [198]. Studies have researched the biological detoxification of mycotoxins; however, there is a lack of research regarding its potential in food and beverage field. Assaf et al. [199,200] have presented an innovative device that might be used to remove mycotoxin from beverages. The machine has probiotic LAB biofilms attached to a special cartridge; as a result of allowing liquids to flow through these adsorbents, the liquid is detoxified. The use of lactic acid bacteria and certain species of yeast that may remove mycotoxins from foods like beer, wine, and fruit-based beverages is acceptable to consumers [45]. Microbial control techniques may degrade the product quality by absorbing nutrients and releasing metabolites into the food chain, despite the fact that biological control is environmentally friendly and possess health attributes. However, mycotoxin reduction is effective and faster when compared with other methods.

Biocontrol of fermented foods and beverages has generated considerable interest as a promising low-cost, natural, and safe option for quality and safety assurance that meets current consumer requests for clear labels and minimally processed foods. It implies using microbial cultures and/or their enzymes or antimicrobial metabolites, to prevent or limit fungal growth or for mycotoxin detoxification purposes through binding to cell walls or degradation into less or nontoxic compounds [198,201,202]. The broad spectrum of food applications, due to the existing wide variety of bacteria and yeasts, with biopreservation features, has been highlighted among the advantages of this approach [201]. Yet, although certain microorganisms have been shown to reduce mycotoxins bioavailability, they still cannot absorb/destroy them completely [198]. Thus, the application of microbial consortiums represents a potential method for an efficient co-degradation of multiple mycotoxins [203].

Table 2 summarizes recent investigations concerning the application of biological decontamination assays in fermented beverages. Similar studies in different food matrices have also been performed and reported elsewhere [204].

Further research is needed to elucidate the specific mechanisms of mycotoxin binding/degradation, including toxicology studies of metabolites, and for the establishment of optimum conditions for the application of these processes in large-scale industrial settings. The development of recombinant degrading enzymes, capable of degrading multi-toxins, should also be prioritized [192]. In parallel, the suitability of using combined strains for different food applications and mycotoxins mixtures must be explored, focusing on improving the efficacy of detoxification. Furthermore, the physicochemical, sensorial, and nutritional profile of fermented beverages, after mycotoxin detoxification, must be assessed [187,190,192].

## 6. Final Remarks

The market of fruit-based fermented beverages includes innovative and traditional drinks that has gained consumers’ appreciation due to their unique and surprising sensorial features and positive impact on human health. On the other side, reducing fruit byproducts and wastes may be accomplished by developing new fermented beverages. Regardless of the raw materials state (i.e., whole, part of a fruit, byproduct, waste) entering the production process, the final product may be subject to biological, physical, or chemical risks. Risk causes may have multiple and variate sources; maximum levels may vary and are regulated under different legislations, but reducing or, if possible, eliminating any hazard is in the spotlight of any national and international authority and food producer. Thus, informing and awareness of any potential risk that may contaminate the fruit-based fermented beverages is the first step in developing any procedure meant to increase food safety.

It is of paramount importance that the manufacturers know the techniques used to limit the contaminants’ formation and remove them. One of the most considerable effects of biotechnology on the food industry has been the use of enzymes, which is a successful technique for resolving processing problems and practically enhancing the quality and safety of fruit-fermented drinks. Frequently exploited decontamination techniques use microorganisms able to adsorb, in their cell walls, mycotoxins from the dietary matrix and make them less or non-toxic. Alcoholic beverage detoxification from mycotoxins may be performed using chemical treatments that include hypochlorite treatment, ammoniation, ozone treatment, alkaline hydrogen peroxide treatment, chemical adsorbents, and food additives.

We underline the importance of informing the producers of the potential risks that can endanger the safety of fruit-based fermented beverages and their awareness of the techniques to reduce or eliminate them.

## Figures and Tables

**Table 1:** Application of removal techniques for chemical contaminants in fermented fruit-based beverages.

Risk Category	Contamination Level	Method or Technology	Thermal/Non-Thermal Procedure	Removal Yield	Reference
Biogen amine	Histamine concentration: 0.01–0.07 mg/20 mL wine	Ultrasonic treatment at 50 Hz along with 0, 10, and 30 mg naringenin	Non-thermal procedure	The sample treated at 50 Hz + 30 mL naringenin for 30 min showed a maximum reduction in the histamine concentration of 79.52%	[187]
	Histamine concentration: 0.1 mL of bacterial inoculum of each HIS/10 mL wine ~10^6^ CFU/mL	Sustainable and lightweight graphene aerogel (GA)	Non-thermal procedure	A percentage of 80% of HIS (cell counts produced by bacterial contaminants: *Cronobacter sakazakii*, *Staphylococcus aureus*, and *Aeromonas* sp.) was extracted from red wine after 60 min under acidic (3.0) and neutral (7.4) pH conditions, using the synthesized GA	[188]
	MRS broth supplemented with tyramine mmol/L and putrescine 1 mmol/L	*Lactobacillus plantarum*	Non-thermal procedure	Twenty-four hours after the inoculation of the *L. brevis* IOEB 9809 and *E. faecium* OT23 the concentration of putrescine and tyramine was lowered to 29.62 and 38.17%, respectively.	[189]
Pesticides	Pyrimethanil, Vinclozolin Cyprodinil, Procymidone concentration for each pesticide: 34 µg/L, 28 µg/L, and 51 µg/L	Pulsed electric field (PEF)	Thermal procedure	The results were directly proportional to the strength and energy of the treatment For cyprodinil, concentrations decreased by 18%, 36%, and 48%, respectively. For procymidone, there was a similar decrease of 16%, 15%, and 23%. Vinclozolin followed the same trend with decreases of 4%, 15%, and 32%, and pyrimethanil by 2%, 14%, and 27%.	[190]
	Tebuconazol, Thiamethoxam concentration for each pesticide 10, 20, 30, 40, 50, 60, 70, 80, 90, 100 mg/L	Ultrasonic treatment	Non-thermal procedure	The removal rate of pesticides was between 72.1% and 100%	[191]
	Fungicides, Insecticides, Herbicides concentration for each pesticide 0.1, 0.9, 2.3, mg/L	Microfiltration	Non-thermal procedure	According to the reduction effectiveness of the pesticides, the membranes were range: for white wine, as: CA > CN > PESU > NY > RC > PA and red wine: CA > CN > RC > PESU > PA > NY	[192]
	Microorganisms in the vineyard: fungal contamination 15.3 CFU/g leaf bacteria contamination 40.7 CFU/g leaf yeast contamination 18 CFU/g leaf	Ozone (O_3_)	Non-thermal procedure	Treatment with ozonated water resulted in a reduction of fungal infestation to 8.0 CFU/g leaf, while, surprisingly, bacteria and yeasts had higher CFU levels on treated leaves, to 54.7 CFU/g leaf and 39.3 CFU/g leaf, respectively	[193]
	Chlorpyrifos, Ethion, Diazinon, Fenitrothion, Fenthion, Phorate. contamination 200 μg/kg grape	Chitosan as fining agent	Non-thermal procedure	The efficiency of pesticide removal by chitosan ranged from 54% to 72% at a chitosan concentration of 0.05% and increased to 86 to 98% when using a higher chitosan concentration (max 0.5%) in comparison to other clarifiers, 0.05% chitosan had the greatest pesticide removal efficiency (72%)	[194]
Mycotoxins	Microbial contamination: *Saccharomyces cerevisiae* concentration of 7.4 log_10_ CFU/mL	Non-thermal high voltage atmospheric coldplasma (HVACP)	Non-thermal procedure	HVACP treatment of grape juice at 80 kV for 4 min resulted in a reduction of 7.3 log_10_ CFU/mL of *S. cerevisiae* without considerable (*p* > 0.05) change in pH, acidity, and electrical conductivity of the juice	[195]
	Ochratoxin A (OTA) concentration 5000 ng/L	Alginate-PVA-*L. plantarum*	Non-thermal procedure	The APLP complexes were effective in removing OTA from wines without significantly influencing their phenolic quality. A time of 52 min was required to achieve the goal of removing over 50% of the OTA	[160]
	Ochratoxin A (OTA) concentration 10 μg/L	Activated carbons (ACs)	Non-thermal procedure	In white wine, OTA was completely removed, whereas red wine had a 40% removal efficiency	[196]
	Patulin (PAT) contamination 1.0 mg/L	UV light	Thermal procedure	The UV fluence that leads to more than 70% reduction of patulin. However, the UVC lamp (222 nm) was the most effective UV source reducing 90% of PAT. No significant changes in pH, total soluble solids, and color in apple juice after UV exposure	[162]
	Alternariol (AFB1) and Aflatoxins B1(AOHB1) concentrations of 100 µg/L	HPP	Non-thermal procedure	Treatment increased AFB1 and AOH removal by 24% and 37%, respectively, compared with thermal treatment in the different models studied	[197]

**Table 2:** Selected recent studies (2020–2022) regarding biological decontamination in fermented beverages.

Fermented Beverages	Biocontrol Strain	Inhibited/Detoxified Microorganism/Mycotoxin	Removal (%)	Reference
Apple cider	*Saccharomyces cerevisiae* 1027	Patulin	20.8–49.1	[205]
Fermented apple juice	*Lactobacillus pentosus* DSM 20314	Patulin	53.14	[206]
Fermented apple juice	*Lactobacillus plantarum* 13M5	Patulin	53.6	[207]
Fermented grape juice	*Lacticaseibacillus rhamnosus* 6133	Ochratoxin A	35.34	[208]
Red wine	*Oenococcus oeni*	Histamine	NA	[209]
Wine-like medium	*Saccharomyces eubayanus* NPCC 1302	Yeast	NA	[210]

NA: not applicable.

## Data Availability

Not applicable.
